# Fractures du bassin occasionnant une plaie du vagin

**DOI:** 10.11604/pamj.2021.38.70.22402

**Published:** 2021-01-21

**Authors:** Kouamé Innocent M´Bra, Kouamé Jean Eric Kouassi, Bada Justin Leopold Niaore Sery, Loukou Blaise Yao, Aya Adélaïde Natacha Kouassi, Yao Aboh Ganyn Robert Arnaud Asséré, Pierre Germain Jr Ochou, Régis Akobé, Koffi Léopold Krah, Michel Kodo

**Affiliations:** 1 Service d´Orthopédie et de Traumatologie du CHU de Bouaké, Bouaké, Côte d´Ivoire

**Keywords:** Fractures du bassin, plaie du vagin, traitement, Pelvic fractures, vaginal wound, treatment

## Abstract

Les fractures du bassin surviennent le plus souvent au décours d´un traumatisme violent. Malgré ce contexte de haute vélocité, ces lésions sont rarement associées à des plaies du vagin et les données sur l´évolution sont rares. L´objectif de notre étude était de décrire les lésions anatomocliniques, le traitement et l´évolution de ces lésions. Nous avons observé cinq cas de plaies du vagin au cours d´une fracture du bassin chez la femme au cours de ces dix dernières années. Les patientes avaient un âge moyen de 23,6 ans. Le motif principal était les accidents de la voie publique. Deux patientes présentaient des plaies linéaires et trois présentaient des plaies délabrantes. Des sutures vaginales ont été réalisées chez toutes les patientes. Au recul moyen de deux ans, l´évolution a été favorable avec cicatrisation de la plaie vaginale et de l´os. Les activités génitales et obstétricales n´ont pas été compromises. Ce sont des lésions qui passent le plus souvent inaperçues. Il faudra y penser devant tout traumatisme du bassin chez la femme.

## Abstract (en)

Pelvic fractures occur most often in people experiencing a traumatic event. Although they are due to high-velocity injuries, these lesions are rarely associated with vaginal wounds, and data on patients’ evolution are scarce. The purpose of our study was to describe anatomoclinic lesions, treatment and progression of these lesions. The study involved five female patients who had had vaginal wounds due to pelvic fracture over the past decade. The patients had a mean age of 23,6 years. Vaginal wounds were mainly due to road accidents. Two patients had linear wounds and three had lacerated wounds. Vaginal suture was performed in all patients. After a mean follow-up period of 2 years, patients’ evolution was favorable with healing of vaginal wound and bone. Genital and obstetric functions were not compromised. Although vaginal wounds most often go unnoticed, they should be suspected in female patients with pelvic fracture.

## Introduction

Les fractures du bassin sont considérées d´emblée comme graves car surviennent le plus souvent dans un contexte de polytraumatisme, au décours d´une chute de lieu très élevé, d´un accident de la voie publique qui sont généralement de haute vélocité et l´incidence rapportée est de 8 à 15% [1,2]. Les fractures ouvertes du bassin demeurent rares et plus encore lorsque l´ouverture se situe à l´intérieur du vagin chez la femme [3,4]. Cette faible incidence pourrait être le fait que ces fractures passent inaperçues à cause des contractions du muscle pelvien en réaction au stress post-traumatique qui contribue à arrêter les saignements et aussi à cause de l´importance des autres lésions qui masquent le diagnostic. De même ces saignements pourraient être considérés à tort comme des menstrues. L´objectif de notre étude était de décrire les lésions anatomocliniques, le traitement et l´évolution de ces lésions.

## Méthodes

Il s´agit d´une étude rétrospective sur une période de dix ans 2009 à 2019 au cours de laquelle nous avons diagnostiqué et traité cinq patientes présentant une fracture du bassin avec une lésion vaginale sur 304 fractures du bassin au cours de la même période. Nous avons étudié les paramètres épidémiologique, diagnostique, thérapeutique et évolutif des fractures du bassin avec plaie vaginale.

## Résultats

Cinq patients ont été diagnostiqués et traité au cours de cette période. La moyenne d´âge était de 23,6 ans (18-40 ans). Score de gravité des blessures était de 27 (10-48), la moyenne de temps mis du lieu de l´accident aux urgences était de 2,8 h (1h et 5h). Toutes les lésions ont été causées par des accidents de la voie publique. Il s´agissait d´une patiente piétonne qui a été renversée par une voiture et la roue avant lui serait passée sur le bassin et la cuisse. Chez deux patientes, il s´agissait de passagères à bord chacune dans des véhicules qui ont fait une sortie de route. Elles auraient reçu le siège avant sur le pubis. Chez deux patientes, il s´agissait de passagères arrière de moto qui ont fait une chute avec réception sur le bassin. Les signes cliniques étaient marqués par des saignements abondant à la vulve chez deux patientes et des saignements discrets chez trois patientes. Toutes les patientes étaient hors de la période menstruelle au moment de l´accident. Les différentes explorations ont montré que les saignements provenaient du vagin. Deux patientes présentaient des plaies linéaires et trois présentaient des plaies délabrantes au niveau du vagin. Chez deux patientes on percevait un fragment osseux au toucher à travers la plaie vaginale. Au plan radiographique, il s´agissait de 2 cas de fracture tile A et 4 cas de fracture tile B. Aucune patiente n´a présenté de lésion de la vessie ni de l´urètre. Aucun signe de choque hémodynamique n´a été enregistré. Une réparation des plaies vaginales a été faite par suture chez toutes les patientes et un patch imbibé d´antiseptique a été placé à chaque fois dans le vagin. Le traitement des fractures a été fait par repos sur plan dur pendant 21 à 30 jours. Au plan génital, on n´a pas observé d´infection. La cicatrisation s´est faite en une moyenne de 21 (14 et 28) jours. Au plan osseux, la réparation a été acquise après une moyenne de 120 (90 et 150) jours. Une patiente a développé un syndrome de morel-lavallée à la région trochantérienne, ce qui a été traité par incision évacuatrice. Aucune patiente, ni leur partenaire n´ont présenté de plainte lors de rapport sexuels. Trois patientes ont fait des accouchements d´un enfant sain par voie basse à 16 mois, 18 mois et 20 mois après l´accident.

## Discussion

Les fractures du bassin se compliquent généralement de lésions vésicales ou urétrales. Les complications génitales sont rarement rapportées dans la littérature. Cette rareté pourrait s´expliquer par la plaie à l´intérieur du vagin qui rend difficile le diagnostic. Des lésions génito-urinaires basses doivent être systématiquement suspectées chez la femme présentant un traumatisme pelvien avec fracture du bassin [5-8]. L´intérêt donc d´un examen gynécologique au spéculum devant tout saignement vaginal même occulte est nécessaire devant tous traumatisme du bassin. Surtout que l´incidence des fractures du bassin chez la femme est supérieure à l´homme avec un ratio de 1,5/1 [9]. Ces lésions génito-urinaires doivent être surtout suspectées quand il existe un saignement vaginal, retrouvé dans 80% des cas par Perry et Husmann [10] et dans 100% dans notre étude. Ce signe important, ne doit cependant pas être confondu avec la présence de menstruations [10]. Dans nos cas les patientes n´étaient pas en période menstruelle, ce qui a fortement motivé l´examen au spéculum dans l´optique de déterminer l´origine des saignements qui provenaient de la paroi vaginale. Il faut savoir que les signes cliniques périnéaux peuvent ne pas refléter la sévérité des lésions [11]. Le rapport de VENN fait état de trois cas d'avulsion vaginale complète [9]. L'urètre et le vagin étant anatomiquement proches alors le fascia vésico-urétro-vaginal est facilement déchiré. Des cas de lésions associées de l'urètre et du vagin ont été rapportés [7,11-13]. Certains auteurs rapportent des associations de lésions urétrales et rectales [14].

**Figure 1:**
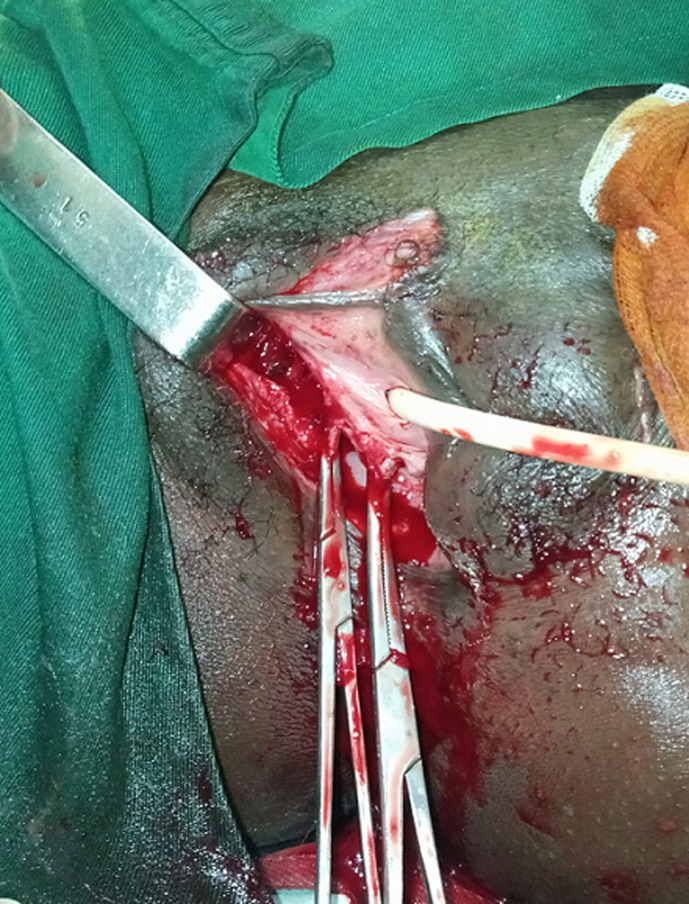
plaie délabrant du vagin

**Figure 2:**
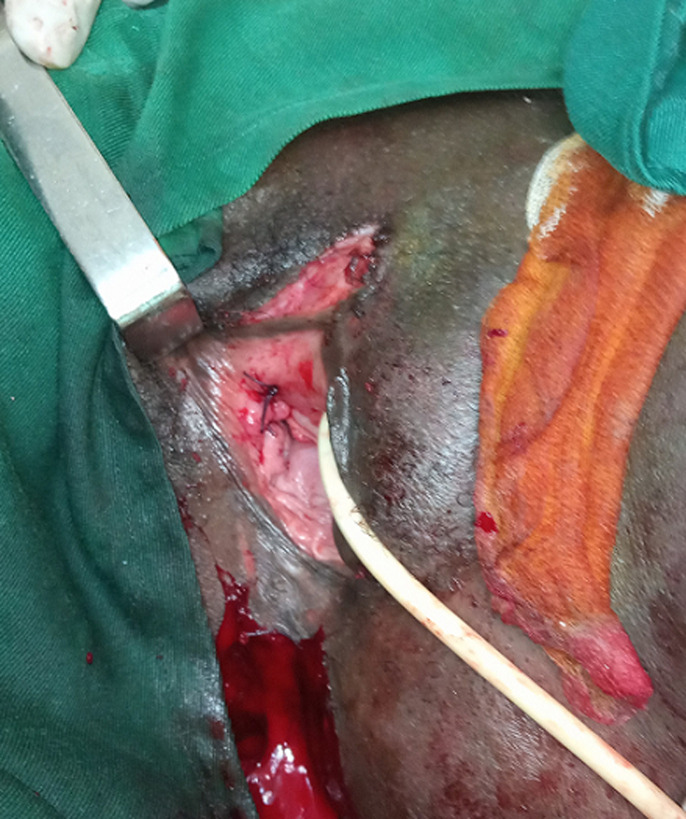
plaie suturée de la paroi du vagin

**Figure 3:**
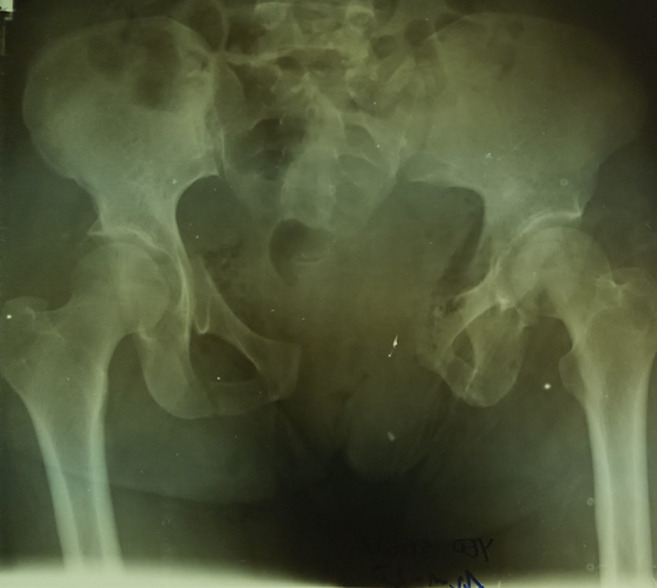
fracture du bassin tile B

Dans nos cas on retrouve des lésions isolées du vagin. Niemi et Norton [3] observaient aussi des plaies vaginales isolées. L´association de ces lésions avec des ruptures urétrales, complique le plus souvent le tableau. Perry et Husmann [10] relatent deux cas (2/6) où, par faute de diagnostic, il a été observé l'apparition d'une fasciite nécrosante puis d'un choc septique sur extravasation d'urine infectée. Dans les cas rapportés ici, les diagnostics ont été précoces autant que les prises en charge, ce qui a contribué à une évolution sans complications aussi bien au plan génital qu´obstétrical sur un recul moyen de deux ans. Niemi et Norton [3] relatent un cas de diagnostic tardif, à 72 heures, ayant évolué vers un important abcès pelvien. En outre ces lésions imposent un suivi qui doit être prolongé afin de déceler des complications tardives qui pourraient survenir à type de fistule urétro-vaginale, d´infection génitale ou d´ostéite dont le traitement demeure long et difficile. Certains auteurs rapportent une corrélation entre le type de fracture et le haut risque de lésion vaginale [4,15,16]. Nous avons rencontré de façon prépondérante le type B de tile et 1 cas de type A. Des études ont montré que les fractures du bassin ne contre indiquent pas des accouchements par voie basse. Dans notre série les patientes ont accouché d´un enfant sain par voie basse sans aucune difficulté (Figure 1, Figure 2, Figure 3).

## Conclusion

Il faut savoir toujours penser à une lésion vaginale devant toutes fractures du bassin chez la femme et surtout dans le cas de polytraumatisme où la fracture elle-même passe le plus souvent inaperçue. Il serait donc important de réaliser un examen gynécologique à la recherche de lésion vaginale dont la méconnaissance pourrait engager le pronostic vital.

### Etat des connaissances sur le sujet

- Ce sont des lésions qui passent le plus souvent inaperçues;
- Les données sur l´évolution sont rarement rapportées.

### Contribution de notre étude à la connaissance

- Ces lésions ne compromettent pas le pronostic de l´accouchement par voie basse;
- Ces lésions ne compromettent pas la vie génitale.
